# Minimal change disease in horseshoe kidney

**DOI:** 10.11604/pamj.2017.26.243.11438

**Published:** 2017-04-28

**Authors:** Yosr Chaabouni, Rahma Guesmi, Yosr Hentati, Khaoula Kammoun, Mohamed Ben Hmida, Zeineb Mnif, Tahya Boudawara, Jamil Hachicha

**Affiliations:** 1 Department of Nephrology, Hedi Chaker Hospital, Sfax Tunisia; 2 UR12ES14, Faculty of Medicine, Sfax Tunisia; 3 Department of Radiology, Hedi Chaker Hospital, Sfax, Tunisia; 4 Department of Anatomopathology, Habib Bourguiba Hospital, Sfax, Tunisia

**Keywords:** Horseshoe kidney, nephritic syndrome, glomerulonephritis, minimal change disease

## Abstract

The horseshoe kidney is a frequent urological birth defect. The most frequent complications are urinary tract infections, stones and hydronephrosis. The occurrence of glomerular disease in horseshoe kidney is rare. Therefore, we report the first case of minimal change disease occurring in a patient with horseshoe kidney in literature. A 22-year-old Caucasian man without personal or family medical history admitted to the pneumology department for a pulmonary artery embolism. In presence of a generalized oedema, a biological assessment was performed yielding intense nephrotic syndrome with urine protein excretion 22g/day. The abdominal ultrasound revealed a horseshoe kidney. Hence a scanno-guided kidney biopsy was taken yielding minimal change disease. High dose steroids were started, then gradually tapered with good response. Horseshoe kidney is the most common renal fusion anomaly, with a prevalence of 0.25% among the general population. The occurrence of glomerular nephropathy in horseshoe kidney has been reported in few cases. We report the first case of minimal change disease occurring in a patient with horseshoe kidney in literature. The mechanism of the association between the horseshoe kidney and these renal pathologies could not be explained in the previous reports. There is no literature data indicating a high rate of glomerulonephritis in horseshoe kidneys. The co-incidence of two renal diseases in this patient can be only a coincidence. The question that arises is whether this glomerulopathy is associated or not with this anatomical abnormality. Further studies are needed to answer this question.

## Introduction

The horseshoe kidney (HSK) is a frequent urological birth defect, with a prevalence of 0.25% among the general population [1]. The most frequent complications are urinary tract infections, stones, hydronephrosis and a variety of benign and malignant tumors [1, 2]. The occurrence of glomerular nephropathy in HSK has been only reported in a few case reports of patients with membranous glomerulonephropathy [3-5], focal and segmental glomerulosclerosis [2, 6, 7], membranoproliferative glomerulonephritis [8, 9], renal amylosis [2], immunoglobulin A nephropathy [10] and a Henoch-Schonlein purpura nephritis [10]. We report the first case of minimal change disease (MCD) occurring in a patient with HSK.

## Patient and observation

A 22-year-old man without personal or family medical history admitted to the pneumonology department for a pulmonary artery embolism.In physical examination, blood pressure was 120/70mmHg, pulse was 110/min, and temperature was 37,5°C. A thoracic computed tomographyangiography objectified a proximal left pulmonary embolism with a focus of infarction and left pleural effusion.The heparin therapy is then given followed by an overlap with vitamin K antagonists. In presence of a generalized oedema, a biological assessment was performed yielding intense nephrotic syndrome with 24 hours urine protein excretion 22g/day. Prior to transfer to the nephrology division, the patient had a massive hemoptysis with respiratory distress secondary to overdose of vitamin K antagonists (INR 8,15). There fore he was transferred to the intensive care unit. Anticoagulant treatment was stopped, he received vitamin K infusions and transfused with fresh frozen plasma. Two days later, his condition has stabilized and heparin was re-introduced. Therefore, he was transferred to the nephrology division. Physical examination on admission, showed: blood pressure was 110/60mmHg, pulse 85/min, weight 97kg, generalized oedema, urine test rendered (++++) proteinuria and (-) hematuria. The laboratory tests showed the following results: haemoglobin 11,3g/dl,total protein 36g/l, serum albumin 12,9g/l, blood urea nitrogen 6,5mmol/l, serum creatinine 95μmol/l, total cholesterol 8,14mmol/l, triglycerides 3,13mmol/l, serum immunoglobulin levels and complement levels (C3, C4) were in the normal range. Hepatitis B surface antigen, anti-HCV antibodies and immune deficiency virus antibody were all negative. The blood coagulation function of the patient was normal. Abdominal ultrasonography and computed tomography, detected the presence of HSK, the right kidney was in the lumbar region and the left one is in the pelvic region, with no other abnormalities.

Before renipuncture, the patient had signed informed consent after he was informed of the significance and risks of renipuncture, moreover, percutaneous scanno-guided renal biopsy was performed by experienced doctors, on the right renal upper pole, using a standard needle biopsy gun. The patient did not present any postoperative complications. Biopsy material, examined by light microscopy (PSA stain), showed 9 glomeruli, with no extra- capillary proliferation, no endocapillary proliferation, no thickening of the basement membranes, no interstitial fibrosis and no deposit on the immunofluorescence stain, concluding to MCD. Proteinuria declined gradually than disappeared over 4 weeks with prednisolone and angiotensin-converting enzyme inhibitor (150mg/day, Lopril), and oedema disappeared. Prednisolone treatment was started with an 80 mg dose for 4 weeks and then was gradually diminished. Vitamin K antagonists were stopped after 3 months.

## Discussion

HSK is the most common renal fusion anomaly, with a prevalence of 0.25% among the general population. It consists of kidney fusion across the midline. HSK can be present as an isolated condition in 30%, but there are a wide variety of associated abnormalities. The most frequent include ureteropelvic junction obstruction, lithiasis, infections and a variety of benign and malignant tumors [1, 2, 11]. There is no literature data indicating a high rate of glomerulonephritis in HSK. However, the occurrence of glomerular nephropathy in HSK has been only reported in a few cases including membranous glomerulonephropathy [3-5], focal and segmental glomerulosclerosis [2, 6, 7], membranoproliferative glomerulonephritis [8, 9], renal amylosis [2], immunoglobulin A nephropathy [10] and a Henoch-Schonlein purpura nephritis [10] (Table 1). We are the first to describe the case of MGD occurring in a HSK. Renal structure of HSK is abnormal; moreover, relationship between HSK blood vessels and the adjoining great vessels is complex, which may increase both the difficulty and the riskassociated with performing renipuncture [12]. The HSK is one of the main indications for transjugular renal biopsy [13]. Renal biopsy may be valuable and viable for HSK patients with heavy proteinuria to identify pathologic type of glomerulopathy and to guide treatment, if renal biopsy is performed by experienced doctors at the renal upper pole under renal ultrasonic guidance.

**Table 1:** Published data on glomerular diseases occurring in horseshoe kidneys

Authors	Sex	Age (years)	Serum creatinine	Urine protein	Pathological findings
Chen A et al, 1990 [3]	M	20	0,9mg/dl	300mg	Membranous glomerulonephropathy
Abson C et al, 1991 [6]	M	52	1,5mg/dl	7,7-14,4g/24h	Focal and segmental glomerulosclerosis
Fujimoto S et al, 1992 [4]	M	48	0,7mg/dl	0,6g/24h	Membranous glomerulonephropathy
Matyus J et al, 1996 [9]	M	38	210μmol/l	9g/24h	Membranoproliferative glomerulonephritis
Alagözlü H et al, 2001 [5]	F	18	0,8mg/dl	8-14g/24h	Membranous glomerulonephropathy
Kavukcu S et al, 2003 [8]	M	8	0,8mg/dl	50mg/m^2^/h	Mesangioproliferative glomerulonephritis
Kayatas et al, 2007 [2]	M	23	0,9mg/dl	9,2g/24h	Focal and segmental glomerulosclerosis
Kayatas et al, 2007 [2]	M	28	0,8mg/dl	5,8g/24h	Renal amyloidosis
Rivera et al, 2010 [7]	M	38	1,6mg/dl	18g/24h	Focal and segmental glomerulosclerosis
Hu P et al, 2014 [10]	F	15	66,9 μmol/l	1,7g/24h	Henoch-Schonlein purpura nephritis
Hu P et al, 2014 [10]	M	26	108,2 μmol/l	1,4g/24h	Immunoglobulin A nephropathy

The mechanism of the association between the HSK and these renal pathologies could not be explained in the previous reports. HSK is a structural and developmental anomaly. Wilms tumor has been reported to be seen four times more in this group of patients [1]. In addition, chromosomal abnormalities like Turner syndrome and trisomy 18 are also seen more commonly in patients with HSK [14]. Glomerular pathologies have not been reported frequently in these chromosomal diseases or Wims tumor. Furthermore, glomerulonephritis incidence has not been reported to increase in structural and developmental anomalies of the kidney like dysplasia or hypoplasia. HSK differs from the other anomalies and from the normal kidney with respect to its size and blood supply. Further studies are needed to identify the relationship between HSK and glomerulopathy.

## Conclusion

This case of MCD indicates that the response to treatment of glomerulonephritis in HSK does not differ from the glomerulonephritis in normal kidneys. In addition, the mechanism of the association of HSK with glomerulonephritis should be evaluated and screened further, keeping in mind that it could just be coincidental. The question that arises is whether this glomerulopathy is associated or not with this anatomical abnormality. Further studies are needed to answer this question. Finally, awareness of embryology and anatomy is essential to assess and understand the complications affecting HSK.
